# The Use of Empirical Methods for Testing Granular Materials in Analogue Modelling

**DOI:** 10.3390/ma10060635

**Published:** 2017-06-09

**Authors:** Domenico Montanari, Andrea Agostini, Marco Bonini, Giacomo Corti, Chiara Del Ventisette

**Affiliations:** 1 Institute of Geosciences and Earth Resources, National Research Council of Italy (CNR), 50121 Florence, Italy; keisi79@yahoo.it (A.A.); mbonini@geo.unifi.it (M.B.); giacomo.corti@cnr.it (G.C.); 2 Earth Sciences Department, University of Florence, 50121 Florence, Italy

**Keywords:** brittle material properties, analogue experiments, friction, cohesion, sand mixture, Hausner ratio, empirical methods, density

## Abstract

The behaviour of a granular material is mainly dependent on its frictional properties, angle of internal friction, and cohesion, which, together with material density, are the key factors to be considered during the scaling procedure of analogue models. The frictional properties of a granular material are usually investigated by means of technical instruments such as a Hubbert-type apparatus and ring shear testers, which allow for investigating the response of the tested material to a wide range of applied stresses. Here we explore the possibility to determine material properties by means of different empirical methods applied to mixtures of quartz and K-feldspar sand. Empirical methods exhibit the great advantage of measuring the properties of a certain analogue material under the experimental conditions, which are strongly sensitive to the handling techniques. Finally, the results obtained from the empirical methods have been compared with ring shear tests carried out on the same materials, which show a satisfactory agreement with those determined empirically.

## 1. Introduction

Analogue modelling represents one of the most useful tools to test hypotheses on the evolution and controlling factors of geological processes at different length and time scales. One of the fundamental steps is to identify the proper analogue material to replicate natural rocks and model the natural process under investigation.

Although the behaviour of natural rock is complex (i.e., experimentally deformed rocks are characterized by an elastic/frictional plastic behaviour with strain hardening prior to failure, and strain softening after failure; e.g., [1] and references therein), the simple Coulomb rheology may be successfully used to describe the mechanical behavior of upper brittle crust on a regional scale [2]. This can be justified from the observation that rocks are affected by faults and fractures of any dimensions and orientation, coupled with mechanical heterogeneities of different origins that cause failure by reactivation to occur before the peak strength (undeformed rock strength) is reached.

The most common type of failure at the upper brittle crustal condition (low pressure up to a few hundred MPa) is thus frictional shear failure, governed by the Mohr-Coulomb criterion of failure (Equation (1)) describing the linear increase in the strength of rocks with depth:

(1) τ=μσ(1−λ)+C

where *τ* and *σ* are the shear and normal stress acting on the fracture plane, *C* and *μ* are respectively the cohesion and friction coefficient [3,4], and *λ* is the pore fluid factor, given by the ratio between the fluid pressure and the lithostatic load [5]. On the basis of experimental tests, Byerlee [6] proposed that in pre-fractured rocks the shear stress required to slide rock surfaces over one another decreases linearly as a function of the normal stress according to the relationship *τ* = 0.85*σ* for normal stresses ≤200 MPa, and *τ* = 0.6*σ* + 0.5 for normal stresses >200 MPa.

Following the above concepts, analogue modellers commonly use different dry granular materials (i.e., sand and powders of different composition and granulometry, flour, rice powder, plaster) to simulate upper crustal deformation. Their behaviour is generally assumed to follow the Coulomb failure criterion, with constant frictional properties and is strain-rate independent (e.g., [7]), thereby implying that these materials can properly simulate the brittle rock behaviour. However, laboratory experiments on granular materials show that they exhibit a more complex behavior [8,9,10,11]. For example, the friction coefficient of granular materials is not constant, but strain dependent, showing different values at peak strength (i.e., at fault initiation), stable dynamic strength (fault sliding), and stable static strength (fault reactivation; [8,9]). In addition, Ritter et al. [12] found that strain weakening at normal loads <1 kPa is due to the partial loss of cohesion. Furthermore, for very low stresses (<400 Pa) the failure envelope of granular material is not linear, and thus it is not strictly following the Coulomb criterion [10,13]. Additionally, the main material parameters (density, cohesion, internal friction coefficient μ = tanΦ, where Φ is the angle of internal friction) depend primarily on grain shape, dimensions, and grain size distribution (e.g., [9,10,14,15,16,17,18]), and, secondly, material properties are strongly dependent on the handling technique, i.e., the way they are used in the model building procedure, as this influences the grain packing and the degree of grain compaction (e.g., [9,19,20,21]). For example, the angle of internal friction may vary approximately up to 3°–4° [21]. Nevertheless, the granular material behaviour is very similar to the experimentally deformed rock (e.g., [1,22,23]).

The main material parameters that are necessary to be known for characterizing a granular material for analogue modelling purposes are: (1) the frictional parameters (internal friction coefficient μ or the angle of internal friction Φ, and the cohesion *C*) and (2) the bulk density.

The internal friction coefficient reflects the way that single grains slide against each other [24]. The internal friction coefficient of crustal rock, obtained from laboratory strength tests, varies between 0.5 and 1.0 (angle of internal friction from 26.5° to 45°; [3,25]). The scaling procedure requires its value to be identical—or close—in both the analogue granular material and nature [26]. Consequently, granular materials used in the laboratory usually possess an internal friction angle included in this range (e.g., [9,10,11,17]). Higher or lower values are found only for materials employed to simulate specific conditions, such as for microbeads showing an angle of internal friction as low as 20°. The coefficient of friction is lower for spherical particles, more suitable in modelling brittle behavior in analogue experiments [10], and higher for angular grains [10,14].

Cohesion describes the amount of shear stress that a material can resist in a zero normal stress condition [24]. In non-cohesive granular materials, the interaction between the grains is mainly related to the steric repulsion and to the friction forces. Therefore, the macroscopic properties of the assembly are governed by the geometry of the grains (shape and size distribution) and by the surface properties of the grains [27]. In nature, cohesion is generally in the range of 10^7^ Pa for intact rocks (e.g., [3]); this value may decrease one or two orders of magnitude in strongly fractured rocks ([7] and references therein). Cohesion has the dimension of stress, and thus it must be scaled down by the stress scaling factor, which is the ratio between stress in the model and in nature σ* = ρ*g*h*, where the asterisk denotes the model to nature ratios. Common stress ratios are around 10^−5^–10^−6^ (e.g., [7,28]), and thus it follows that analogue materials should have very low cohesion, on the order of a few to few hundreds of Pa. This requirement is met by many granular materials used in analogue models. More cohesive analogue materials have cohesion of some hundreds of Pa (e.g., gypsum in [20]), but most common values are in the range of 20 Pa to 80 Pa (e.g., [9,10,16,29,30]). However, the measurement of cohesion in granular material is a controversial subject and different contradictory solutions have been proposed. Usually, cohesion of granular materials is inferred from the interception with the shear axis (in the *τ* − *σ* plot) of the straight line obtained by a linear regression of shear test results. However, this method may give values that are not fully realistic since it contradicts the common observation that dry granular materials are virtually cohesionless [29]. Furthermore, if the test is made under very low normal stresses (<400 Pa), the Coulomb failure envelope is not linear but instead it shows a convex-outward shape [10] suggesting that the linearly extrapolated values of cohesion at normal stresses below 400 Pa are overestimated.

Similarly to the angle of internal friction, cohesion shows a lower value for spherical and/or finer particles and higher values for angular and/or coarser material [10,14,16,19,27,31].

The bulk density *ρ* is simpler to be analyzed, nevertheless it is central in the analogue modelling scaling procedure (i.e., *σ* = *ρgh*); if one of the principal stress directions is vertical, the stress system is Andersonian [32] and the failure criterion can be written, in terms of principal stresses, as *σ*_1_ − *σ*_3_ = *αρgh*, where *α* is a parameter dependent on the frictional properties of the material and on the orientation of the stress field [4]. Despite the easiness of the measure, the bulk density is strongly influenced by the handling techniques: differences in density due to the handling techniques range between 5% and 20% depending on the materials [9,19]. Common bulk density values for granular analogue material range between ~400 kg/m^3^ for lighter materials (e.g., diatomite powder; [28]) and ~1900 kg/m^3^ for heavier materials like corundum sand (e.g., [9]).

Generally, the above described analogue material parameters are measured using different apparatuses (i.e., Hubbert-type apparatus [33] and ring shear test [34]) which allow for investigating the response of the tested material to a wide range of applied stresses. However, as stated before, the main parameters are dependent on the handling technique. For this reason, in this paper we apply empirical methods to determine the parameters of granular materials under conditions commonly occurring in experimentral analogue modelling. To test the empirical approach, some measurements have been carried out through the ring-shear tester at the Engineering Geology Laboratory of the University of Florence. Empirical methods have the great advantage of measuring the properties of a given analogue material in the same conditions that the material is utilized in during an experiment.

## 2. Brittle Materials Used in Analogue Modelling

To reproduce in their experiments the brittle crustal rocks, analogue modelers use a wide range of different materials. Each granular material has its own characteristics in terms of the angle of internal friction, cohesion, and density, as well as its grain characteristic that may influence the structural details and the sharpness of the structures. Each material is thus suitable to reproduce a specific geological setting depending on its properties.

Generally, granular materials used in analogue modelling can be divided into two main categories: cohesive and non-cohesive or low cohesion granular materials (e.g., [11,27]). Low cohesion materials are mainly used to model weak materials. The most used low cohesion materials are dry quartz sand (i.e., [9,11,16] and references therein) and glass microbeads (high-quality vaporized glass sphere; [9]). Compared to the other classical granular materials (i.e., quartz sand), glass microbeads are characterized by a lower frictional angle varying between 20° and 29°, [9,35,36,37]. Given their Moho-Coulomb behaviour, microbeads are mainly used to simulate shale and claystone décollement layers and weakness zones within the model (e.g., [9,36,37,38]).

Cohesive granular materials are generally silica powder [39,40], fine-grained ignimbrite powder [41], diatomite powder [28], and gypsum [20]. These materials fail both by tension (open fractures) and shear at low differential stresses, and are widely employed in reproducing volcanic systems and shallow magma intrusion at different scales ([11] and references therein), or for modelling peculiar environments with highly cohesive rocks and predominant open fractures (e.g., [20]).

## 3. Materials and Methods

To obtain new analogue granular materials that would combine the advantages of quartz sand and cohesive materials, we mixed quartz sand and K-feldspar powder in different proportions. Our aim is to obtain a material characterized by a proper internal friction angle able to simulate crustal rocks, with moderate cohesion, as well as a suitable grain size allowing the development of numerous and detailed structures for a complete fracture/fault (statistical) analysis.

We tested quartz sand (Fontainebleau quartz sand, hereinafter simply called Q-sand) and K-feldspar fine sand (Kaolinwerke-AKW feldspar SF 900 SF sand; hereinafter referred as K-feldspar sand) and different mixtures between these two materials (Table 1). The use of this sand mixture should produce in the models a high number of brittle structures with very definite details (see also [13]). This mixture enables the desired analysis without significantly altering the frictional properties and the material density. Sand quantities were weighted with a precision balance and the different mixtures were prepared by mixing the two components in a planetary mixer for at least 5 min to obtain a granulometrically homogenous material.

**Figure 1:**
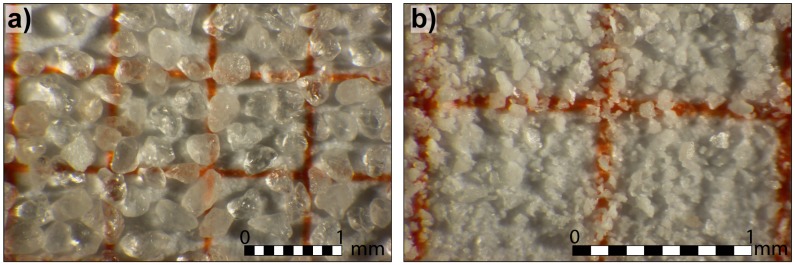
Microscope picture of the pure Q-sand (**a**) and pure K-feldspar sand (**b**). Note the strong differences in terms of grain size, grain size distribution, and grain roundness.

**Figure 2:**
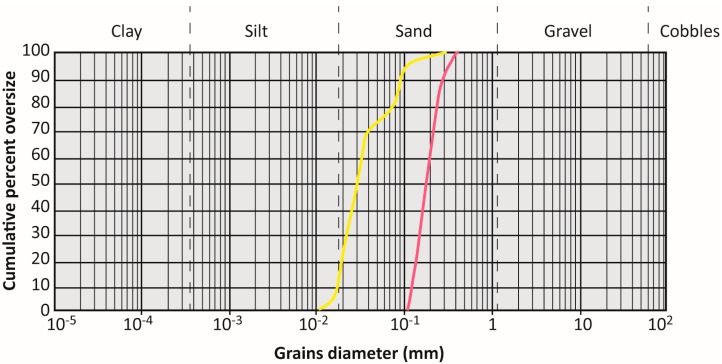
Grain-size distribution curve for the pure Q-sand (**red line**) and the pure K-feldspar sand (**yellow line**).

Fontainebleau Q-sand (Figure 1a and Figure 2) is a well sorted sand (average grain diameter ~200 μm), primarily composed of siliceous and sub-rounded grains. K-feldspar SF 900 SF (Figure 1b and Figure 2) is a poorly sorted sand (grain diameter mostly (90%) in the range of 20–100 μm, with a significant (50%) finer fraction, <30 μm).

The grains have mostly an angular shape, and are mainly composed of potassium feldspar (83%), with subordinate sodium feldspar (6%), quartz (10%), and a smaller part of kaolinite (1%).

As mentioned earlier, granular material properties are strongly sensitive to the handling techniques [8,9,19]: pouring or sifting the same sand results in a change in its frictional properties as well as of its density, because of the different grain packing. For this reason, we handled and prepared the analyzed materials in the same way that we prepare the experiments; particularly, the granular material was carefully poured from a fixed height (i.e., ~10 cm). After the material reaches the desired height (usually a few centimeters), the top surface is scraped off to level the sand and obtain a flat and smooth (initial) surface. This procedure has been demonstrated to not significantly alter the frictional properties of the granular materials [21,42].

### 3.1. Angle of Internal Friction

The angle of internal friction was determined by the means of two different empirical methods widely used in the literature: (1) by measuring the angle of repose [27,43,44,45]; and (2) by measuring the dip angle of faults developed during simple set-up analogue extensional models [9,19], following the principle that the angle θ between the maximum principal stress *σ*_1_ and the new fault plane is given as θ = 45° − Φ/2. 

**Figure 3:**
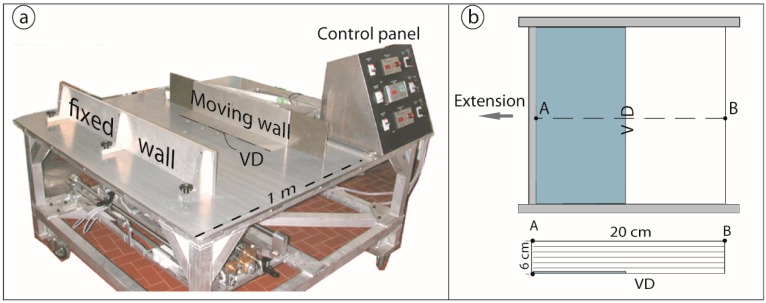
(**a**) Deformation pure-simple shear apparatus (‘Tosi machine’, modified after Montanari et al. [46]); and (**b**) basic model set-up used to evaluate the angle of internal friction.

The experiments were built with a simple set-up (here we are interested only in measuring the materials properties, thus no scaling procedure was adopted): a 6 cm-thick layer of sand mixture with a thin colored layer (made of the same sand mixture) every 1 cm of thickness to help recognizing the faults. The sand is colored with Indian ink and naturally dried, over a period of days. Coloring sand is a very common practice in analogue modeling, and it is recognized to not significantly influence the mechanical properties of sand. The sand pack was 20 cm long and rested on the base of a deforming pure/simple shear apparatus (referred to as the ‘Tosi machine’, at the Tectonic Modelling Laboratory of the Institute of Geosciences and Earth Resources at the Earth Science Department of the University of Florence, Figure 3). Extension was applied by moving a sidewall driven by a stepper motor controlled by a central unit (Figure 3). Half of the model is placed above a thin rigid metal sheet that is connected to the mobile wall, thereby producing a velocity discontinuity (VD) at the metal sheet edge.

Models were slowly stretched (at 2.5 cm/h) and model deformation invariably consisted of a couple of conjugate faults developed above the velocity discontinuity. Extension was applied until the first extensional faults were clearly observable on the mode surface. The deformation distribution at grain size level is sensitive to the material properties, implying that the amount of extension needed for the first faults to develop may slightly vary depending on the model. In any case, the bulk extension was of the order of a few millimeters (2–4 mm). This however does not influence the model results. Once the experiment was stopped, the model was wetted to allow for cutting model cross sections in the model central part. The fault dip was measured directly above the velocity discontinuity, at the model base, where the conjugate faults initiate and have a rectilinear attitude. In this location, the vertical normal stress corresponds to the maximum principal stress *σ*_1_ (e.g., [9]).

The results of these extensional models are used for determining the angle of internal friction Φ (and consequently the internal friction coefficient μ) of the granular materials. Considering that μ is determined by the angle between a fault plane and the direction of the maximum principal stress (θ) by the relationship μ = tan(90° − 2θ), and in the extensional setting (i.e., normal faults) the main principal stress is vertical, then the angle θ is equal to the complementary angle of the fault dip *α* (i.e., θ = 90 − *α*). The value of the internal friction coefficient calculated with the above-described method is sensitive to the handling technique; particularly the more densely packed sifted sand exhibits a higher friction coefficient than poured sand [8,9,19]. Likewise, the coefficient of peak friction calculated from fault dips is higher than the coefficient determined with a ring shear test [9]. We thus paid close attention when preparing the experiments by always following the same procedure: we carefully poured the sand from the same height and, when the desired height was reached, the top surface was shaved to obtain a flat surface.

Alternatively, the angle of internal friction of the tested materials may be derived from the critical angle of repose. The angle of repose Φ of a granular material can be defined as the maximum angle that allows a heap of soil resting on a horizontal surface to remain stable (e.g., [43]), and it is equal to the angle of internal friction (e.g., [27,44]). A non-cohesive granular material or with inter-particles forces negligible compared to gravity (i.e., small cohesion), poured from a single point forms a cone, whose base angle is the angle of repose Φ. Following Coulomb’s criterion, this occurs because the granular media sustain shear stress as in the solid friction (i.e., due to the resistance to movement of one grain over another). The cone is stable until *τ* < μ*σ*, where *τ* and *σ* are the shear and normal stress, and μ is the internal friction coefficient. The slope of the cone is thus limited to a certain slope that is the function of the internal friction coefficient μ (μ and Φ are related by the relation μ = tanΦ).

Many methods have been proposed to measure the angle of repose: static methods, among which are the ‘fixed height cone’, ‘fixed base cone’, and ‘scoop deposition’, and ‘dynamic methods’, like the ‘tilting table’ and the ‘rotating cylinder’. The angle of repose obtained from a static method tends to present a slightly smaller value [47] with the fixed height cone method, as proposed by the American Society for Testing and Materials (ASTM International; [48]), and the Cornforth method (a variation of the fixed base method [49]), giving the best results [43].

**Figure 4:**
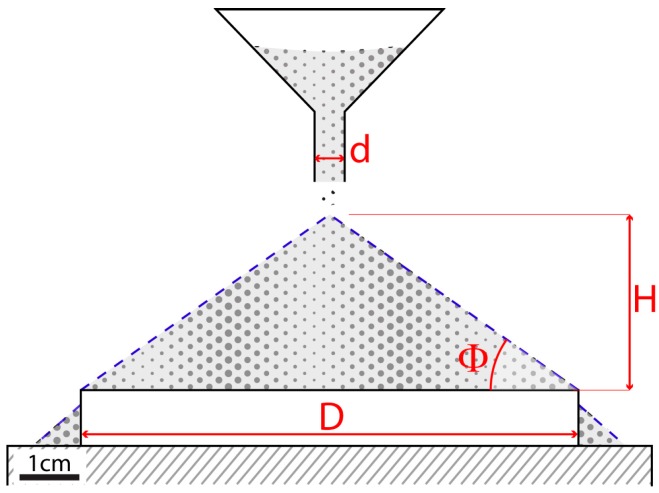
Schematic cartoon showing the measuring procedure of the angle of repose (modified after de Campos and Ferreira [50]).

We thus measured the angle of repose for the five mixtures and the two pure sands with both the fixed height cone method [48] and the fixed base method, which are the most common methods used to determine the angle of repose. Hereafter we shortly describe the two methods used to measure the angle of repose:

‘fixed height cone’ method: the powder is carefully poured through a funnel, at a fixed height until the apex of the heap formed by the granular material reaches the bottom of the funnel (Figure 4); ‘fixed base cone’ method: the granular material flows through a funnel, which is raised vertically until the heap covers a circular base of fixed size. The funnel is slowly raised as the cone grows, maintaining the same distance between the funnel tip and the apex of the cone during the whole procedure.

In both cases, rather than attempt to measure the angle of the resulting cone directly, the angle of repose Φ can be obtained by measuring with a caliper the width of the cone base *D* (86 mm in the fixed base cone method, Figure 4), and the cone height *H* (35 mm in the fixed base cone method); the angle of repose is given by the following equation [47]:

(2) $$\mathrm{angle of repose}=\tan^{-1}\left(\frac{2H}{D-\;d}\right)$$

where *d* is the internal diameter of the funnel nozzle (10 mm).

The fixed base cone method is considered more accurate since *D* is univocally known (while *D* can be ambiguous in the fixed height cone method) [47]. Moreover, in the fixed base cone method, maintaining the same close distance between the tip of the funnel and the top of the growing cone minimizes the impact of falling particles. In both cases, measurements were repeated 10 times and an average value was taken as representative for each mixture. 

In relation to grain size, the pure K-feldspar sand is affected by electrostatic effects [51], and it is not possible to measure its angle of repose by using this method. The high cohesion and the small size of the grains give the pure K-feldspar sand a high angle of repose, and the resulting poor flow properties do not allow the funnel method to be used (the material stagnates inside the funnel and does not freely flow). Also high K-feldspar content mixtures present not optimal flow properties (cfr. [27]); nevertheless, the dimension of the diameter of the funnel outlet accommodates this less well flowing material [47]. However, the influence of the dimension of “*d*” on the angle of repose value is considered in Equation (2) [47].

The angle of internal friction measured with the fault dip methods represents the peak value for the experimental conditions and is a function of the vertical normal stress and thickness of the sand layer (note that the fault angle is measured at the base of the model, at the maximum burial conditions; [9]). Peak values are higher because they are a function of the dilatation that the material should experience before being able to deform and fail (e.g., [8,9]) and may be attributed to the initial yield strength of the sample. Conversely, the angles obtained from the angle of repose correspond to the critical state friction angle or stable friction angle (e.g., [49,52]) linked to the stable sliding (i.e., the state of failure when the stresses do not change anymore during shearing and the volume is constant) (e.g., [8,52]).

### 3.2. Density

The bulk density of a granular material strongly depends on the grain arrangement (and of course on the density of the grains themselves). Density is a function of the grain size, grain shape, and grain size distribution. For example, Klinkmüller et al. [16] established that rounded grains arrange in closer packing than angular grains, when sieved; considering equal grain density and size distribution, rounded materials have a higher bulk density, due to the closer packing. This implies that the density is extremely sensitive to compaction, and that again the handling techniques (i.e., the way we built a model) may influence the density of the used granular materials (e.g., [8,9,19,21]) with a great impact on the whole scaling procedure.

Density was evaluated using an easy and common method: we filled a small container of known volume with the material and we measured its weight with an electronic balance (e.g., [19]). Following the above considerations, we paid close attention while filling the container with the same technique used in preparing the models, specifically the granular material was poured from a constant height of a few centimeters, and once the container was filled, the surface was leveled by scraping with a straightedge. Moreover, to consider the possible compaction of the granular material under its own weight, the container had a height (5 cm) that is a good average value of the model thickness. The obtained density can thus realistically be a good approximation of the density inside the model. At least five measurements were made for each material and averaged.

Successively we proceeded to evaluate how the material packing may influence the bulk density. To obtain an estimate of the compressibility of the analyzed granular material (i.e., the increase in density due to a compression/decrease in volume) we measured the Hausner ratio [53] or compressibility index, which is widely used in the granular material characterisation (i.e., [27,47]). The Hausner ratio is used to quantify the material flow properties, as packing and flow are closely related bulk properties in granular materials [47]. The Hausner ratio is an adimensional number calculated as the ratio between the freely settled bulk density of the granular material and the tapped density, which is an increased bulk density attained after mechanically tapping the container containing the sample. It follows that the greater the Hausner ratio, the greater the bulk density variability due to the different handling technique and the different material packing configurations that may arise. Ideally, a granular material having a low Hausner ratio is an easy to use analogue material (Table 2). To calculate the Hausner ratio we adopted the following common empirical method: 100 g of granular material, weighed with a precision balance, are gently introduced (i.e., with the same method used in building the model) in a graduated cylinder of 250 mL volume, avoiding compaction as much as possible. Knowing the mass (100 g) and the volume V_0_ (from the graduate cylinder), the density D_0_ is calculated. The container is then tapped 500 times on a flat surface. The volume of the granular material after tapping is measured (V_500_), and the tapped density D_500_ is calculated.

The obtained values give a clear idea about the macroscopic properties and the behavior of a granular material [27].

### 3.3. Cohesion

**Figure 5:**
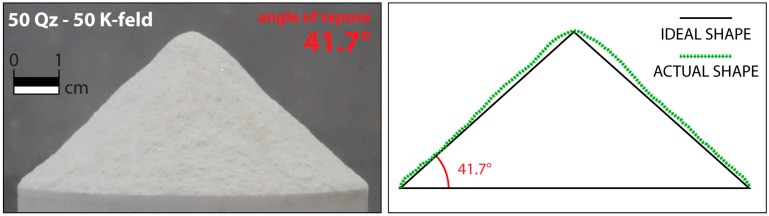
Example of an ideal heap shape compared to the actual heap shape (here for the 50 Quartz—50 K-feldspar mixture). The strong deviation from the ideal shape indicates a high cohesion.

A first qualitative idea of the cohesive properties of a material may arise from the observation of the heap shape obtained by letting the granular material flow through a funnel [27]. The heap shape is strongly dependent on the grain properties. Cohesive granular material gives strong deviations from the conical shape [44]. The isosceles triangle built with the basal angles equal to the angle of repose corresponds to the ideal heap shape (Figure 5). The more the heap shape deviates from the ideal shape, the higher the cohesion; in addition, the cohesive index *σ*_r_ can be obtained from the deviation between the heap interface and the ideal heap shape [27].

A quantitative estimation of the cohesion of a granular material, if cohesion is not negligible, can be made through an empirical method, which is related to the self-sustaining capability of a vertical wall cut in the analyzed material. In this case, Coulomb’s method for wedges [55] predicts that the height of the vertical wall L is limited to the value given by the following equation [44]:

(3) $$L<\frac{4\cos\Phi \times c}{1-\sin\Phi \times \rho g}$$

where *c* is the cohesion, Φ is the angle of internal friction, *g* is the gravitational acceleration, and *ρ* is the material density. From the above equation, it follows that the material cohesion is given by:

(4) $$c=\frac{L_{MAX}\left(1-\sin\Phi \right)\rho g}{4\cos\Phi }$$

This method has been previously used to calculate the cohesion for granular materials used in analogue models (e.g., [7,56,57]). The material was handled in the same way we prepared the experiment, to obtain realistic cohesion values representative of the experimental conditions. This is very important in that in Equation 4 the cohesion is a function of both the density and the angle of internal friction.

Following this method, a thin layer of material was poured on a flat base, and the surface was scraped at a known height and a vertical cut was made in the material with a blade. This process was repeated up to the point where a vertical slope could self-sustain (*L_MAX_*). We repeated this procedure up to 10 times for each material, and an average *L_MAX_* value was obtained for each analyzed material. Since the measurements of the free wall height give very small values (see Section 4.3), pictures of the realized tests were not representative.

## 4. Results of Empirical Methods

### 4.1. Angle of Internal Friction

**Figure 6:**
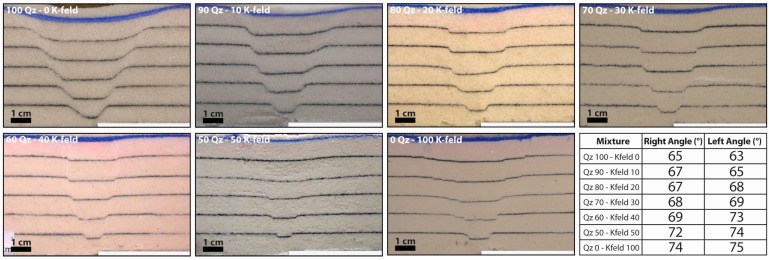
Cross sections of the extensional models used to determine the internal friction angle for the different sand mixtures. The white bar on the lower right angle of each panel indicates the moving metal basal sheet acting as a velocity discontinuity. The table in the right bottom panel summarizes the dip angles of faults on the right and left side of the VD (see text for details).

In this section, initially we show the results obtained through the analysis of faults developed in analog models, and subsequently those obtained considering the critical angle of repose. Fault dip angles are calculated on both the two conjugate faults, the one above the VD (on the right side of the panel in Figure 6; i.e., left dipping) and the conjugate one on the opposite side, which presents slightly different dip values and a curved fault plane (Figure 6). Curved fault planes, with the dip angle increasing toward the shallower levels and becoming almost vertical just below the model surface, are common in these extension experiments. This is because at very low vertical normal stress (i.e., close to the surface) the coefficient of internal friction tends toward infinity and thus the fault dip tends to increase up to 90° [9,10,13]. Similar results in more cohesive materials are explained as a change in the deformation style with depth: tensile failure (i.e., vertical faults) at shallower levels, and shear failure at the base of the model ([20] and references therein). The Table in Figure 6 reports the results of the fault dip test in terms of the angle of internal friction Φ and the coefficient of internal friction μ, calculated as discussed in Section 3.1.

The angle of internal friction has been estimated starting from the condition Φ = 2(45° − θ). However, for each model we have invariably measured slightly different values of θ for the two conjugate normal faults (Figure 6). The different fault dip is presumably related to a reorientation of the vertical maximum principal stress *σ*_1_ above the VD. This variation in fault dip shows a complex and not systematic trend throughout the series of models built with different granular mixtures. (cf. Figure 6). For this reason, in the computation of μ, we have introduced the simplifying assumption of considering the average dip value of the conjugate faults (Table 3).

**Figure 7:**
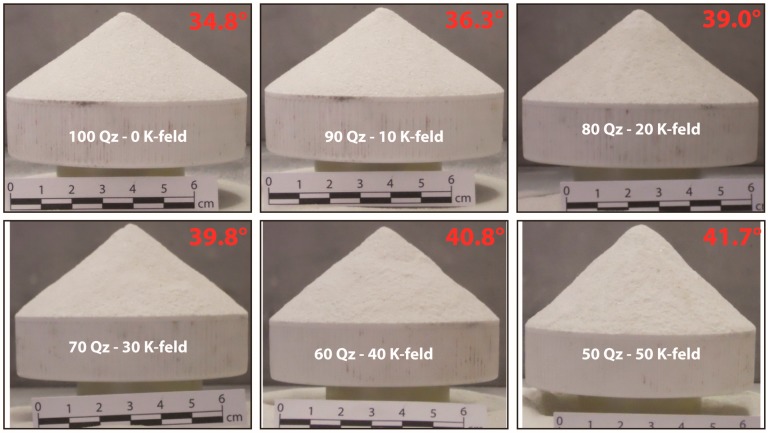
Evaluation of the angle of repose using the ‘fixed base cone’ method. The red number on the right top of each panel indicates the measured angle.

The angle of internal friction is also evaluated using the critical angle of repose through two different approaches, the ‘fixed height cone’ and ‘fixed base cone’ methods. The results obtained with both methods give basically similar results. However, we prefer using the ‘fixed base cone’ method because of its easiness and unambiguity of the measurements (Figure 7 and Table 3).

The angle of internal friction is at a minimum for pure Q-sand and reaches the highest value for pure K-feldspar sand. It increases with the amount of K-feldspar sand in the mixture, showing an almost linear increase (consistent with [16] and [19]).

**Figure 8:**
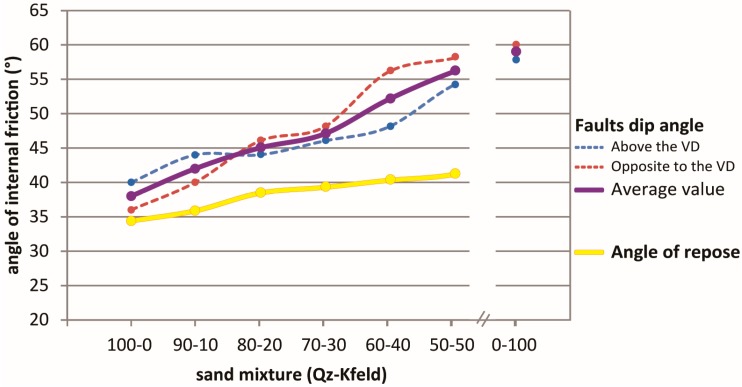
Graph showing the variation of the angle of internal friction as a function of the granular mixture composition as measured with the adopted empirical methods.

As mentioned above, the angle of internal friction when measured with the fault dip methods represents the peak value for the experimental conditions; this explains why this value is always higher with respect to the angle of internal friction calculated considering the critical angle of repose (Figure 8). On the other hand, the lower values of friction angles obtained from the angle of repose correspond to the stable friction angle.

The obtained values for pure Q-sand are well in the range of previous measurements (e.g., [9,27,45,57]). Finally, the high peak values for the mixtures with a higher K-feldspar fraction, measured with the fault dip methods are similar to those of other materials (e.g., [9]).

### 4.2. Density

**Figure 9:**
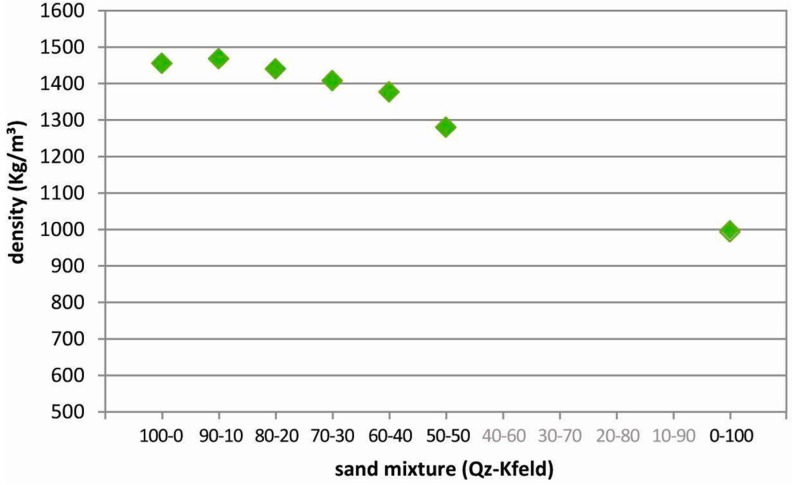
Variation of bulk density as a function of the granular mixture composition.

The obtained values of density are reported in Table 4 and Figure 9. The two pure granular materials used in preparing the different mixtures show a large difference in terms of bulk density at the above-described experimental conditions. Pure Q-sand exhibits a bulk density of 1455 kg/m^3^, while the pure K-feldspar sand has a bulk density of the order of ~1000 kg/m^3^ (the uncertainty in the density value for the K-feldspar sand arises from its characteristics in the flow properties, see above, and compressibility, see below, which do not allow a precise measurement). A progressive increase in the content of the less dense K-feldspar in the mixture should result in a progressive decrease of the mixture bulk density itself. However, we should consider the different grain size of the two pure terms: part of the finest grains of the K-feldspar fill the intergranular voids in between the larger grains of the Q-sand, with no increase in volume. This implies that adding a small quantity of K-feldspar sand to Q-sand results in an increase in the bulk density of the mixture, compared to the pure Q-sand (Table 4 and Figure 9). As a matter of fact, the Qz90-Kfeld10 mixture presents a bulk density that is slightly higher than the pure Q-sand (1468 kg/m^3^). The mixture bulk density tends to slightly decrease with increasing the K-feldspar fraction (>10% in weight); for K-feldspar content higher than 30% in weight, the decrease is more pronounced and tends to become linear.

**Figure 10:**
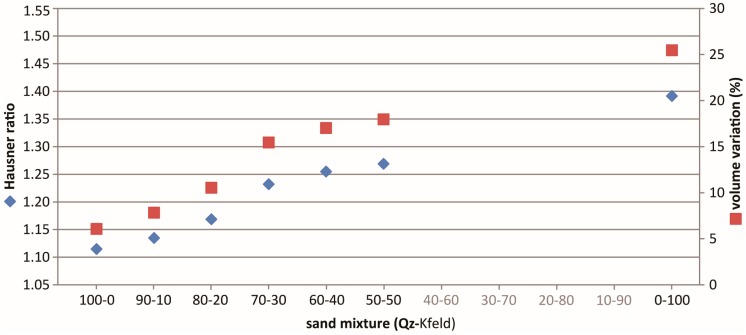
Variation of the Hausner ratio (blue dots) and the volume variation (red dots) as a function of the granular mixture composition.

The Hausner ratio values are shown in Table 5 and Figure 10, together with the volume variation between V_0_ and V_500_. The Hausner ratio (and the volume variation percentage) linearly increases with the K-feldspar content, in a manner comparable with the decrease in density observed above. However, the small increase in density that we observe with small K-feldspar content is not reflected in a small decrease in the Hausner ratio value with an equal mixture composition.

Pure Q-sand, with large, rounded, and poorly-graded grains, is thus characterized by a low Hausner ratio (corresponding to excellent flow properties, Table 2; [27]), which implies small density variation due to the different handling techniques. From this point of view, pure Q-sand is a good analogue material, easy to use, poorly influenced by the handling technique, and that ensures easiness in reproducibility and in the scaling procedure. We should indeed consider that density is a key parameter in the scaling procedure, and that density and the compaction of granular material influence other parameters like the peak friction coefficient and cohesion (i.e., [42]). The pure K-feldspar sand, well-graded and with a conspicuous fine fraction, exhibits a high Hausner ratio (Table 5) which indicates poor flow properties. The analyzed mixtures present an intermediate compressibility index, which corresponds to good (for K-feldspar sand content ≤30%) to fair (K-feldspar sand >30%) flow properties (Table 2). It is thus reasonable to use the mixture with low K-feldspar content (≤30%).

The use of higher K-feldspar mixtures may yield the disadvantage of having dishomogeneities internal to the analogue model, even though high-cohesion materials offer the advantage of suitably reproducing specific geological settings and processes, including those with relatively high length scaling factors (*L_model_*/*L_nature_* ≥ 10^−4^). Moreover, the use of a vibrating device to compact a mixture of different sands could lead to separating the different components, and its use should thus be carefully evaluated when working with sand mixtures.

### 4.3. Cohesion

The angle of internal friction was calculated with two different empirical methods (fault dip method and angle of repose), and the cohesion was accordingly calculated using the two distinct angles of internal friction values (see Equations (3) and (4)) (Table 6). Observing the different shapes obtained by measuring the angle of repose (e.g., Figure 7), it is possible to state that the cohesion is close to zero for pure Q-sand (i.e., the heap shape corresponds to the ideal shape) and it increases with the amount of K-feldspar sand (i.e., the cone shape differs more and more from the ideal shape). For the pure K-feldspar sand (for which it was not possible to obtain a meaningful measure of the repose angle because of the material flow difficulties), the deviation from the ideal shape is even more pronounced, indicating a higher cohesion.

The obtained values are very low, and the cohesion is mostly negligible in the examined granular materials. As for the angle of internal friction, we can observe a clear increase in cohesion with increasing the K-feldspar content in the mixture.

The lower value is found for the pure Q-sand, for which the cohesion is completely neglectable; the observed vertical wall is probably the result of a few grains stacked one on the other and there are no inter-particle forces playing a detectable role. The cohesion obtained from peak friction is maximum for the quartz 60-K-feldspar 40 mixture and then slightly decreases for higher K-feldspar content. The observed increase with K-feldspar is probably related to the higher fine fraction in the mixture, in that the cohesion is the expression of the inter-particle forces and is consequently sensitive to the grain shape and grain size distribution (e.g., [14,44]). Similarly, Krantz [19] observed a slight increase in cohesion by adding clay to sand.

Our results indicate that the cohesion of a granular material is also a function of the density and the degree of compaction [44]. In ccontrast, in their material tests (with a Hubbert-type shear box) Lohrmann et al. [8] found that sand cohesion did not correlate with either sand density or the degree of compaction. Klinkmüller et al. [16] found no significant correlation between cohesion and grain size distribution, and only a strong correlation with grain shape. The measure of cohesion of a granular material and the definition of the influencing factors are thus complex. However, the obtained values are in line with several studies stating that the granular materials, at the stress range of the analogue models, are almost cohesionless, or that their cohesion is very low. Common cohesion values range from zero to a few tens of Pascal, except for high cohesion materials like wet clay or plaster. The main parameters obtained through empirical methods for different sand mixtures are summarised in Table 7.

## 5. Comparison with the Ring Shear Test

**Figure 11:**
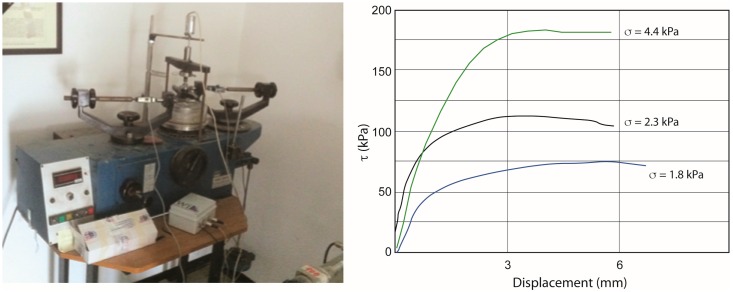
The ring-shear apparatus used in this study and an example of the test results. The curves on the right referr to different applied normal stress ranging from 1.8 kPa (blue line) to 4.4 kPa (green line).

To test the empirical approach, the mechanical properties of different granular mixtures of Q-sand and K-Feld sand were also determined using a ring-shear tester at the Engineering Geology Laboratory of the University of Florence. The apparatus consists of an annular shear cell containing the sample and a lid that is pressed onto the sample at a preset normal load which is kept constant throughout the experiments (Figure 11). Each experiment was performed with a constant deformation rate. Knowing the geometry of the device, one can transform the torque measurements into shear stress (*τ*) and the rotation into displacement and plot those as shear curves. Samples were placed into the shear cell in the most similar way that we used for the empirical methods and analogue models, and measurements were carried out for different normal loads. It should be said, however, that given the conformation of the apparatus, it is not possible to exactly reproduce the same handling techniques and experimental conditions. To test the reproducibility, each measurement was repeated three times per normal load. Ring shear tests were performed at a constant velocity of 0.5 mm/min with a normal stress ranging from 1.8 to 4.4 kPa (shear stress range from 1.3 to 5.6 kPa). For comparison, the results obtained for the pure Q- and K-feldspar sands are reported in Figure 11. The cohesion is inferred from the graphs extrapolating the intersection with the shear stress axis along a straight line. The obtained results are shown in Table 8. Although the ring shear tests show slightly lower angles of internal friction and slightly higher cohesion, there is basically a satisfactorily correlation between these results and those determined empirically.

## 6. Conclusions

To characterise the properties of granular materials used in analogue modelling, modellers commonly use classical technical instruments such as Hubbert-type apparatuses and ring shear testers, which allow investigating the response of the tested material to a wide range of applied stresses. However, analogue modelling laboratories do not always have direct and easy availability of all the equipment needed to carry out these experiments, and above all, these devices often do not allow the characterization of the materials at the experimental conditions, which are affected by particular handling techniques and procedures. In this study, we have explored the possibility to determine the material properties by means of different empirical methods applied to mixtures of quartz- and K-feldspar sand prepared at the experimental conditions. We tested their reliability and we suggested that the adopted empirical methods could represent an easy, cheap, and fast tool for determining the properties of analogue brittle materials, even if, regarding the cohesion evaluation, it is possible that there is an underestimation in comparison to the instrumental measurement techniques that commonly report higher values. Nevertheless, we should consider that by using the most common instrumental techniques (i.e., Hubbert-type apparatus and derived instruments, and ring shear test) and by assuming a linear relationship between the shear stress and normal stress, the cohesion is inferred as the intercept with the shear axis, in a shear stress-normal stress plot. Cohesion values obtained with this method are higher (up to some hundreds of Pa; e.g., [8,19,29,39]) than those we have obtained with the empirical methods. However, for very low normal stresses the relation between the shear stress and normal stress is demonstrably not linear but convex, and the intercept with the shear axis (i.e., the cohesion) is much lower [10]. This suggests that the linearly extrapolated values of cohesion at low normal stresses are overestimated and that at the stress range of the experiments, granular materials should have a very low cohesion. In this view, the results obtained by the empirical approaches are well representative and could provide a feasible and easier way to characterize the properties of granular analogue materials at the experimental conditions.

## Figures and Tables

**Table 1:** Granular analogue materials tested in this study.

Granular Material Name	Q-Sand (% in Weight)	K-Feld Sand (% in Weight)
100 Qz-0 K-feld	100	0
90 Qz-10 K-feld	90	10
80 Qz-20 K-feld	80	20
70 Qz-30 K-feld	70	30
60 Qz-40 K-feld	60	40
50 Qz-50 K-feld	50	50
0 Qz-100 K-feld	0	100

**Table 2:** Empirical relation between the flow properties of a granular material and its Hausner ratio [54].

Flow Property	Hausner Ratio
Excellent	1.00–1.11
Good	1.12–1.18
Fair	1.19–1.25
Passable	1.26–1.34
Poor	1.35–1.45
Very poor	1.46–1.59
Very very poor	>1.60

**Table 3:** Summary table of the angles and coefficients of internal friction of the different granular mixtures measured with the two different empirical methods (fault dip angle and fixed base cone; see text for details).

Mixture (Qz-Kfeld)	Fault Dip Angle				Angle of Repose	
	Φ (°) Right Side (Above VD)	Φ (°) Left Side	Φ (°) Average Value	µ	Φ (°)	µ
100-0	40	36	38	0.73	34.8	0.70
90-10	44	40	42	0.84	36.3	0.74
80-20	44	46	45	1.04	39.0	0.81
70-30	46	48	47	1.11	39.8	0.83
60-40	48	56	52	1.48	40.8	0.86
50-50	54	58	56	1.60	41.7	0.89
0-100	58	60	59	1.73	-	-

**Table 4:** Density values for the different granular mixtures.

Mixture (Qz-Kfeld)	Density (kg/m^3^)
100-0	1455
90-10	1468
80-20	1440
70-30	1408
60-40	1377
50-50	1280
0-100	~1000

**Table 5:** Hausner ratio and volume variation, in percentage, for the different granular mixtures (see text for details).

Mixture (Qz-Kfeld)	Hausner Ratio	Volume Variation (%)
100-0	1.07	6.1
90-10	1.09	7.8
80-20	1.12	10.4
70-30	1.19	15.6
60-40	1.21	17.0
50-50	1.22	17.9
0-100	1.35	25.5

**Table 6:** Cohesion of the different mixtures measured with the maximum vertical wall methods (see text for details). The two different values (1 and 2) arise from the different angles of internal friction used in the calculation (Equations (3) and (4)).

Mixture (Qz-Kfeld)	*L_MAX_* (mm)	Density (kg/m^3^)	Angle of Internal Friction Φ (°)		Cohesion (Pa)	
			1. Fault Dip Angle (Peak Friction)	2. Angle of Repose (Stable Friction)	1. Peak Friction	2. Stable Friction
100-0	0.5	1455	38	34.8	0.9	0.9
90-10	1.25	1468	42	36.3	2.0	2.3
80-20	3.75	1440	45	39.0	5.5	6.3
70-30	6.5	1408	47	39.8	8.8	10.5
60-40	10	1377	52	40.8	12.2	16.2
50-50	11.5	1280	56	41.7	11.0	16.2
0-100	16.5	1000	59	-	11.9	-

**Table 7:** Main parameters obtained through empirical methods for the different sand mixtures.

Mixture (Qz-K-Feld)	Angle of Internal Friction (°)		Density (kg/m^3^)	Cohesion (Pa)		Hausner Ratio	Volume Variation (%)
	Peak Friction	Stable Friction		Peak Friction	Stable Friction		
100-0	38	34.8	1455	0.9	0.9	1.07	6.1
90-10	42	36.3	1468	2.0	2.3	1.09	7.8
80-20	45	39.0	1440	5.5	6.3	1.12	10.4
70-30	47	39.8	1408	8.8	10.5	1.19	15.6
60-40	52	40.8	1377	12.2	16.2	1.21	17.0
50-50	56	41.7	1280	11.0	16.2	1.22	17.9
0-100	59		~1000	11.9		1.35	25.5

**Table 8:** Comparison between the angle of internal friction and cohesion values (peak friction) obtained with the ring shear test and with adopted empirical methods.

Granular Material	Standard Geotechnical Test		Empirical Method	
	Angle of Internal Friction (°)	Cohesion (Pa)	Angle of Internal Friction (°)	Cohesion (Pa)
100 Qz-0 K-feld	33	17	38	0.9
0 Qz-100 K-feld	53	37	59	11.9
